# Phenotypic Changes Exhibited by *E. coli* Cultured in Space

**DOI:** 10.3389/fmicb.2017.01598

**Published:** 2017-08-28

**Authors:** Luis Zea, Michael Larsen, Frederico Estante, Klaus Qvortrup, Ralf Moeller, Sílvia Dias de Oliveira, Louis Stodieck, David Klaus

**Affiliations:** ^1^BioServe Space Technologies, University of Colorado Boulder, Boulder CO, United States; ^2^Department of Biomedical Sciences, University of Copenhagen Copenhagen, Denmark; ^3^Department of Aerospace Engineering Sciences, University of Colorado Boulder, Boulder CO, United States; ^4^Space Microbiology Research Group, Department of Radiation Biology, Institute of Aerospace Medicine, German Aerospace Center Cologne, Germany; ^5^Immunology and Microbiology Laboratory, The Pontifical Catholic University of Rio Grande do Sul Porto Alegre, Brazil

**Keywords:** microgravity, bacterial growth, cell size, cell envelope, vesicle, aggregation, bioastronautics

## Abstract

Bacteria will accompany humans in our exploration of space, making it of importance to study their adaptation to the microgravity environment. To investigate potential phenotypic changes for bacteria grown in space, *Escherichia coli* was cultured onboard the International Space Station with matched controls on Earth. Samples were challenged with different concentrations of gentamicin sulfate to study the role of drug concentration on the dependent variables in the space environment. Analyses included assessments of final cell count, cell size, cell envelope thickness, cell ultrastructure, and culture morphology. A 13-fold increase in final cell count was observed in space with respect to the ground controls and the space flight cells were able to grow in the presence of normally inhibitory levels of gentamicin sulfate. Contrast light microscopy and focused ion beam/scanning electron microscopy showed that, on average, cells in space were 37% of the volume of their matched controls, which may alter the rate of molecule–cell interactions in a diffusion-limited mass transport regime as is expected to occur in microgravity. TEM imagery showed an increase in cell envelope thickness of between 25 and 43% in space with respect to the Earth control group. Outer membrane vesicles were observed on the spaceflight samples, but not on the Earth cultures. While *E. coli* suspension cultures on Earth were homogenously distributed throughout the liquid medium, in space they tended to form a cluster, leaving the surrounding medium visibly clear of cells. This cell aggregation behavior may be associated with enhanced biofilm formation observed in other spaceflight experiments.

## Introduction

By default, bacteria will accompany humans in our exploration of space. The average healthy individual carries trillions of microorganisms in and on their body, outnumbering human cells (Sender et al., 2016). This human microbiome includes opportunistic pathogens, microbes that do not normally cause disease in a healthy person but can provoke an infection when the person’s immune system is suppressed, a concern known to occur during spaceflight (Borchers et al., 2002; Mermel, 2013). It is therefore important to understand bacterial behavior in space in preparation for future long-term human space exploration missions. Numerous prior studies performed in space have shown increased bacterial virulence and decreased susceptibility to antibiotics for select *in vitro* cultures with respect to Earth controls (Tixador et al., 1985, 1994; Lapchine et al., 1986; Moatti et al., 1986; Klaus and Howard, 2006; Wilson et al., 2007, 2008; Parra et al., 2008; Kitts et al., 2009; Ricco et al., 2010). While bacteria are generally considered too small to be directly affected by the reduced gravity of spaceflight (termed “microgravity” for being close to 10^-6^ g), it is hypothesized that they are indirectly impacted by changes in the fluid boundary layer surrounding the cell, as extracellular mass transport becomes essentially limited to diffusion due to the lack of gravity-driven convective flows (Klaus, 1994). Our group recently published a related molecular genetic study indicating that non-motile bacterial cells cultured in liquid medium in space experienced a lack of substrates and increased acidity in their local environment relative to the bulk fluid, which further supports this altered extracellular transport model (Zea et al., 2016).

To characterize related potential phenotypic changes to bacteria cultured in the microgravity environment of spaceflight, *Escherichia coli* was sent in stasis to the International Space Station (ISS) and cultured for 49 h, with matched controls maintained under 1 *g* conditions on Earth. Previous spaceflight studies have presented mixed results in terms of phenotypic expression, including altered envelope thickness and cell aggregation (Zaloguyev et al., 1984; Tixador et al., 1985, 1994; Gasset et al., 1994; Menningmann and Heise, 1994; Juergensmeyer et al., 1999). Based on these studies, it was hypothesized that cells in space would present an increase in cell envelope—which can have implications for drug resistance—and would form aggregates in the absence of disrupting sedimentation motion. Furthermore, it was hypothesized that, in space, cells would grow in the presence of otherwise inhibitory antibiotic concentrations. To test this, seven concentrations of gentamicin sulfate (from 25 to 175 μg/mL in 25 μg/mL increments) were added to *E. coli* cultures in space, but only the three lowest concentrations were evaluated in Earth controls, since it was already known that the normal minimum inhibitory concentration was reached at this point under the ground test conditions. Thus, this experiment was designed with two independent variables: drug concentration, with no-drug samples as controls, and gravitational regime, with Earth samples as controls. The no-drug samples—both on Earth and space—were requested to be fixed too early in the experiment, therefore their data, while not completely lost as it provided insight into the state of the cultures at drug introduction, did result in the loss of the direct no-drug controls. However, the multiple data sets still enable comparison of samples independently in each separate gravitational regime as a function of varying drug concentration, as well as comparison of the samples cultured in space with respect to their matched Earth controls. Furthermore, Brown–Forsythe and Welch’s statistical analyses indicated where it was possible to aggregate data from samples with different drug concentrations to enable space vs. Earth comparisons. This study presents observations made in regards to changes in bacterial growth, cell size, cell envelope thickness, cell ultrastructure, and culture morphology observed in space with respect to matched Earth controls, and as a function of varying drug concentration in each environment.

## Materials and Methods

**FIGURE 1:**
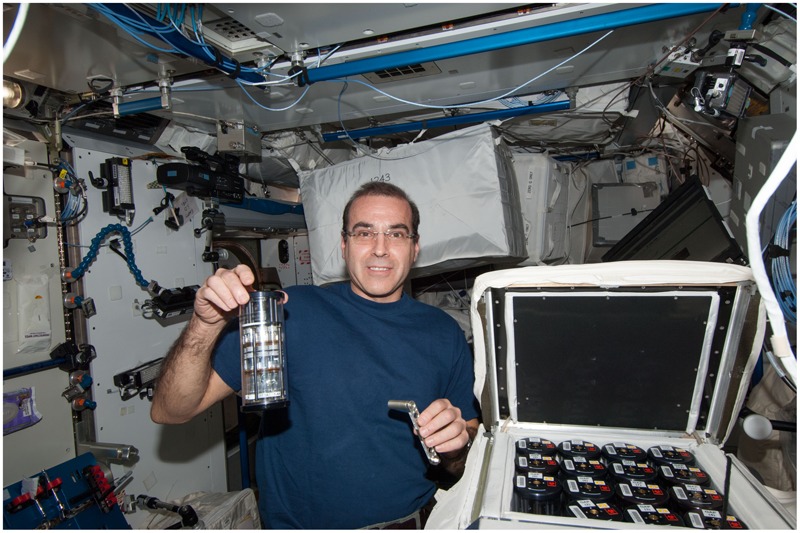
NASA Astronaut Rick Mastracchio shown holding a GAP in his right hand and the crank used to perform an activation in the other as he performed the experimental operations onboard the International Space Station. The open CGBA incubator loaded with other 15 GAPs is in the bottom right. Image: NASA. The individual in this manuscript has given written informed consent to publish this image.

The experiment design, bacterial model, growth medium and temperature, antibiotics, fixative, hardware, sample preparation and loading, operational timeline, and cell count methodology are described in detail in Zea et al. (2016), a related article from the same study, and are summarized here. Briefly, 44 spaceflight samples were used for the analysis reported in this manuscript. Seven concentrations (25–175 μg/mL in 25 μg/mL increments) of gentamicin sulfate (MP Biomedical, Cat No. 1676045, Santa Ana, CA, United States) were assessed in quadruplicates (7 × 4 = 28 samples). At the end of the experiment, samples were fixed with a final 1.5% paraformaldehyde (PFA) (ACROS, Cat. No. 41678, NJ, United States) solution in PBS (Fisher 506 Scientific, Cat. No. TA-125-PB, Waltham, MA, United States) (pH 7.0) as fixative for post-flight phenotypic analyses. The remaining 16 spaceflight samples had no antibiotic and were used as controls. These sets were requested to be fixed too early in the experiment to be used in the final cell count, but they helped reveal cell concentrations at experiment start and at the time of antibiotic introduction. Twenty Earth control samples were used for the analysis reported in this manuscript. The lowest three concentrations of gentamicin tested in space were assessed on Earth in quadruplicates (3 × 4 = 12 samples). The highest four concentrations were not prepared for the Earth controls samples since it had already been determined during pilot studies that bacterial growth was inhibited by this point. Earth controls were also fixed with 1.5% PFA in PBS. The remaining eight Earth samples contained no antibiotics and were used as controls as described above. *E. coli* ATCC 4157 (non-motile) was used for this experiment, cultured in Medium E minimal growth medium (Vogel and Bonner, 1956) supplemented with 5g/L glucose (Fisher Scientific, Cat. No. D-16, Waltham, MA, United States) at 30°C, as this temperature allows for a clearer differentiation of the growth phases (Kacena et al., 1999; Klaus and Ahmed, 2010). Earth samples were cultured statically (i.e., not shaken) to avoid introducing a confounding factor. Spaceflight and Earth samples were cultured in identical hardware and growth conditions: BioServe’s Fluid Processing Apparatus (FPA) in Group Activation Pack (GAP) (Hoehn et al., 2004). In space, they were inside the Commercial Generic Bioprocessing Apparatus (CGBA) incubator as shown in **Figure 1**. Cell count was measured with a hemocytometer post-flight, as the reduction in cell size in space with respect to Earth controls made the results from a plate reader inaccurate.

### Operational Timeline

The timeline for the preparation of the media and cultures, integration of the samples into the Cygnus spacecraft, berthing to the ISS and experiment activation is described in Zea (2015) and Zea et al. (2016). The first experimental operation (*t*_0_) mixed the inoculum with the growth medium contained in the FPA (with the exception of the samples used to assess cell count at this time where the inoculum was mixed with fixative). The second operation, which occurred 19 h later (*t*_1_ = *t*_0_ + 19 h), mixed the antibiotic solution with the bacterial culture (with the exception of the samples used to assess cell count at this time where fixative was mixed with the culture instead of antibiotics). The final operation occurred 30 h later (*t*_2_ = *t*_1_ + 30 h), introducing the fixative into the bacterial culture. Thus, the total incubation period, maintained at 30°C, was 49 h. The protocols were implemented identically for the space and matched Earth sets.

### Minimum Inhibitory Concentration for Spaceflight Operations

The operational constraints associated with conducting spaceflight research required incorporating a modified version of the standard minimum inhibitory concentration (MIC) protocol described by Andrews (2001). The pre-flight (1 *g*) baseline MIC determination consisted of challenging an *E. coli* inoculum in a dilution series of gentamicin sulfate using glass test tubes that replicated the FPAs. After inoculating the antibiotic dilution series, samples were incubated anaerobically for 32 h and growth was assessed via photometry at that point. The MIC was determined to be in between the lower drug concentration where growth was still measured after incubation and the upper concentration where no growth was observed. As indicated in **Supplementary Figures S1, S2**, the MIC as tested was close to 20 μg/mL of gentamicin sulfate for the conditions specific to this spaceflight experiment.

### Statistical Analyses

Welch’s test is the statistical tool used to test the hypothesis that two populations with unequal variances and samples sizes have equal means (Welch, 1947). Because of this flexibility, this statistical test was used to compare (a) cell growth in space vs. Earth at the time of experiment start and (b) of antibiotic introduction, (c) final cell count on the three lowest drug concentrations, (d) cell length, (e) cell diameter, (f) cell volume, and (g) cell envelope thickness on the two lowest antibiotic concentrations, as all of these sets compared two groups with unequal variances and sample sizes. Brown–Forsythe is a statistical test that transforms the response variable allowing to perform a one-way ANOVA on groups with unequal variances (Brown and Forsythe, 1974). This test was chosen to assess the role of drug concentration on cell (a) length, (b) diameter, and (c) envelope thickness at each gravitational environment (microgravity and 1 g), since these groups had different sample sizes and variances.

### Phase Contrast Microscopy and Cell Size Measurements

Since the samples were already fixed in 1.5% PFA, no sample preparation was required for microscopy, which was used for analyzing cell and colony morphology, and cell length and diameter. Samples were not centrifuged or otherwise manipulated prior to light microscopy. Phase contrast microscopy was performed using a Carl Zeiss Axio Imager M2 and a Nikon E600 Widefield Microscope. Cell length and diameter data were acquired using ZEN (Zeiss, 2014) and FIJI (LOCI, 2014) software. Cell length and diameter data were acquired only from cells with their axis coplanar to the microscopy image, i.e., only of cells that were “lying flat” with respect to the image. Cell volume was calculated by modeling a bacterial cell as a cylinder with two hemispheres.

### Transmission Electron Microscopy

Following centrifugation, the supernatant was replaced and the sample pellets re-suspended and rinsed in 0.15 M sodium cacodylate buffer (pH 7.2), three times. Next, the sample pellets were embedded in low-melting point agarose and postfixed in 1% w/v OsO_4_ in 0.12 M sodium cacodylate buffer (pH 7.2) for 2 h. The specimens were dehydrated in graded series of ethanol, transferred to propylene oxide and embedded in Epon according to standard procedures. Sections, approximately 80 nm thick, were cut with a Leica Ultra microtome UC7 and collected on copper grids with Formvar supporting membranes, stained with uranyl acetate and lead citrate, and subsequently examined with a Philips CM 100 TEM (Philips, Eindhoven, Netherlands), operated at an accelerating voltage of 80 kV. Digital images were recorded with an OSIS Veleta digital slow scan 2k × 2k CCD camera (Olympus, Germany) and the ITEM software package.

### Focused Ion Beam/Scanning Electron Microscopy

The Epon embedded specimens were placed in a Dual-Beam microscope (Quanta FEG 3D FIB-SEM, FEI, Netherlands) equipped with a gallium ion source for milling and a dedicated backscattered electron detector for imaging (vCD). The surface of the block and the trimmed edge was located with the secondary electron detector in standard SEM mode with definition of an area of interest. For focused ion beam milling the block was tilted to 52° and the edge of the block aligned at eucentric height, followed by crossover alignment of both electron and ion beams. The ion beam was used in conjunction with a gas injection system to deposit a 1 μm layer of platinum on the top surface of the sample, above the region of interest to reduce milling artifacts. Next, trenches approximately 6 μm wide were milled at high beam current on both sides of the region of interest to avoid deposition artifacts. The G2 Slice and View software (FEI) was used for automatic milling and image recording with automatic refocusing of the exposed surface.

### 3D Image Reconstruction

Digital image datasets were recorded from the sample blocks containing space flight 25 μg/mL (790 images) and Earth control 25 μg/mL sets (748 images). The images with the specifications of 2048 × 1768 pixels in 8 bits were assembled and automatically aligned in Amira 6.0.0—ResolveRT. Voxel size was *x* = 6.7 nm, *y* = 8.5 nm, and *z* = 30 nm. From the two image stacks, all bacteria were automatically masked using the LabelField and all well-fixed and non-edematous bacteria were included for 3D reconstruction. Identical mask parameters for the two samples were manually set in the masking dialog. 3D models were generated by the SurfaceGen and projected by the SurfaceView. Finally, the images were captured in high resolution and post-produced in Photoshop CC.

## Results

### Bacterial Growth

**FIGURE 2:**
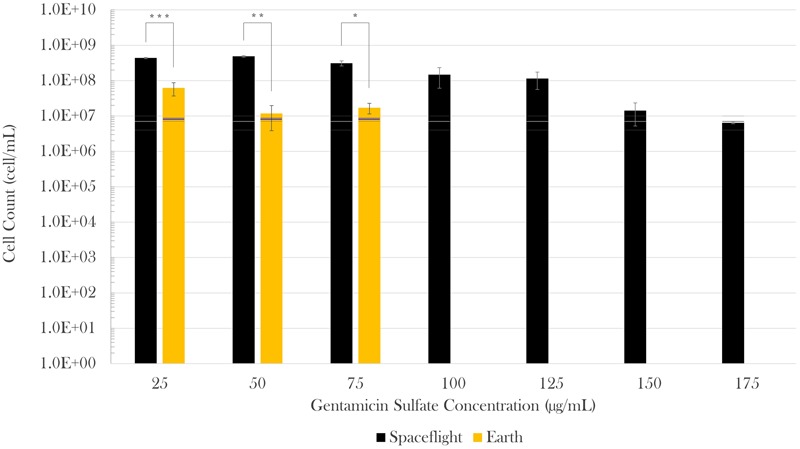
*Escherichia coli* growth challenged with gentamicin sulfate. Black bars represent spaceflight; gold bars are Earth samples. The gray and blue lines indicate the cell concentration at the time of antibiotic introduction in space and Earth, respectively, showing the average (center line) and standard errors (upper and lower lines). Spaceflight cultures showed higher cell counts than their Earth matched controls (13-fold increase in average). Although there appears to be a decrease in magnitude at 150 and 175 μg/mL with respect to 125 μg/mL, this is misleading as accurate values were hard to acquire either by cell count or optical density due to cell aggregation in these samples. It is estimated that values at 150 and 175 μg/mL were roughly equivalent to that of 125 μg/mL. Bars indicate standard error; *n* = 4 for all except for spaceflight at 25 and 50 μg/mL (*n* = 3, each), and 175 μg/mL (*n* = 2), as only the samples for which it was certain that the antibiotic was fully introduced were considered. ^∗^*p* ≤ 0.05, ^∗∗^*p* ≤ 0.01, and ^∗∗∗^*p* ≤ 0.001.

Cell concentration in the spaceflight and Earth control samples was measured post-flight via hemocytometer count by fixing samples at three different time points in the experiment: (1) start (inoculation), (2) acceleration phase (in between lag and exponential phases, when the antibiotic was introduced), and (3) at the end of the test (stationary phase). The spaceflight and Earth cell counts were compared with Welch’s tests since they had different sample sizes and variances. No statistically significant difference was observed in cell count at experiment start (*M*_space_ = 3.75 × 10^5^ cell/mL, *SE*_space_ = 1.34 × 10^5^ cell/mL, *n*_space_ = 8; *M*_Earth_ = 1.25 × 10^5^ cell/mL, *SE*_Earth_ = 7.22 × 10^4^ cell/mL, *n*_Earth_ = 4) or acceleration phase (*M*_space_ = 6.9 × 10^6^ cell/mL, *SE*_space_ = 2.8 × 10^6^ cell/mL, *n*_space_ = 8; *M*_Earth_ = 7.9 × 10^6^ cell/mL, *SE*_Earth_ = 8.3 × 10^5^ cell/mL, *n*_Earth_ = 4) in between spaceflight and matching Earth controls. Spaceflight cell concentrations at the end of the experiment, however, were higher than their respective Earth controls in all cases. When challenged with 25 μg/mL of gentamicin sulfate, there was a sevenfold increase in final cell count on spaceflight with respect to Earth [Welch’s *F*(1,4.82) = 156.83, *p* < 0.001]. This increase was 41-fold [Welch’s *F*(1,2.37) = 300.69, *p* < 0.01] and 18-fold [Welch’s *F*(1,3.07) = 32.96, *p* < 0.05] for the next two antibiotic concentrations (50 and 75 μg/mL), respectively. Conducting a Welch’s test on these three lowest concentrations as a single group showed that there was a 13-fold increase in final cell count in space with respect to Earth [Welch’s *F*(1,10.88) = 116.68, *p* < 0.0001]. **Figure 2** describes the differences in cell count in space (black bars) with respect to Earth controls (gold bars), and as a function of drug concentration in each gravitational environment. A Welch’s test conducted to evaluate growth from the time of antibiotic introduction until fixation showed that the flight samples achieved a significant increase in cell count at the lowest three drug concentrations (25, 50, and 75 mg/mL), while the corresponding ground controls did not exhibit any statistical growth difference in that timeframe.

### Cell Size

**FIGURE 3:**
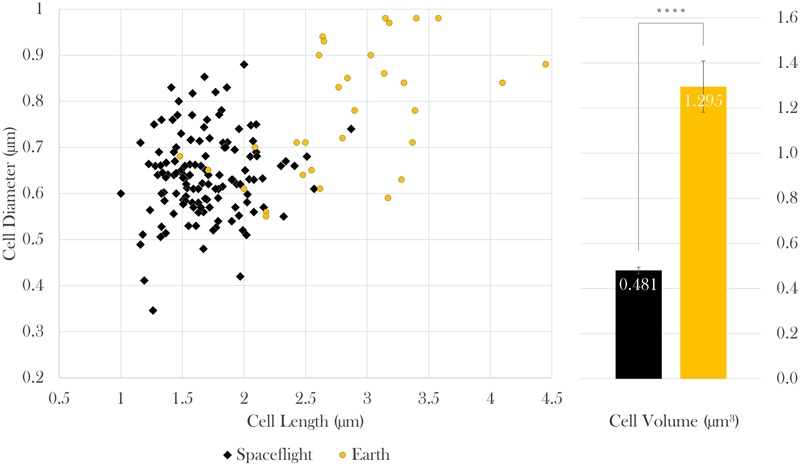
In space, *E. coli* cells were 59% the length, 83% the diameter, and thus 37% the volume of Earth controls. Bars indicate standard error; *n* = 133 and 31 for spaceflight and Earth, respectively. ^∗∗∗∗^*p* ≤ 0.0001.

**FIGURE 4:**
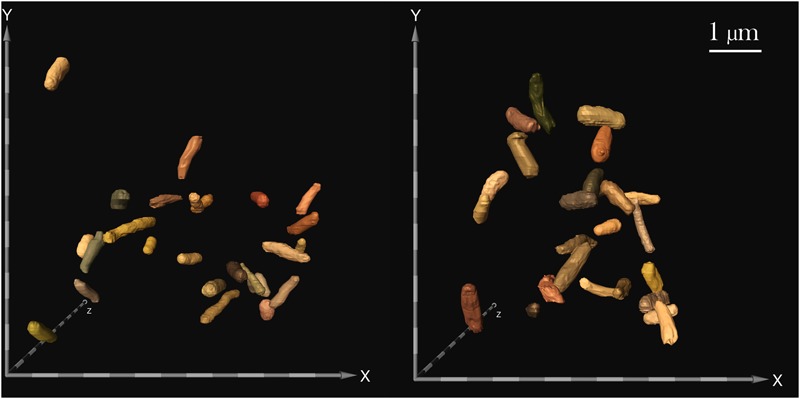
Focused ion beam/scanning electron microscopy (FIB/SEM) image of samples challenged with 25 μg/mL gentamicin: spaceflight (left) and Earth control (right). FIB/SEM analysis corroborated the findings on reduced cell size in space. Scale bar on axes = 500 nm.

Cell size measurements were taken from all samples using contrast light microscopy. The paired cell length and diameter data of each measured bacterium was used to calculate cellular volumes independently. Because sample sizes and variances differed among samples, Brown–Forsythe tests were conducted to assess the role of drug concentration on (a) cell length and (b) diameter at each gravitational environment (microgravity and 1 *g*). It was concluded that neither cell length nor cell diameter were dependent upon drug concentration in space [length: Welch’s *F*(6,17.92) = 2.03, *p* = 0.12; diameter: Welch’s *F*(6,32.48) = 1.78, *p* = 0.14] or on Earth [length: Welch’s *F*(2, 8.71) = 1.73, *p* = 0.24; diameter: Welch’s *F*(2,16.52) = 3.46, *p* = 0.06], which enables comparison of cell size values acquired from space as a collective data set to those from Earth as another data set (**Figure 3**). Two Welch’s tests were performed to determine if the gravitational environment had an impact on cell length or cell diameter. It was found that, in space, cells averaged 59% the length and 83% the diameter of the Earth controls (see **Table 1**). Thus, on average, cells in space were smaller at 37% of the volume of the Earth controls, as indicated in **Figure 3**. Focused ion beam/scanning electron microscopy (FIB/SEM) analyses corroborated the observed reduction in size on cultures grown in space (**Figure 4**).

**Table 1:** Results and statistics of cell length, diameter, and volume of spaceflight and Earth samples; *n*_space_ = 133 cells, *n*_Earth_ = 31 cells.

	Space	Earth	Space wrt Earth	Statistical test	*p*
Length	*M* = 1.684 μm, *SD* = 0.319 μm	*M* = 2.838 μm, *SD* = 0.644 μm	59%	Welch’s *F* (1,33.51) = 94.24	<0.0001
Diameter	*M* = 0.638 μm, *SD* = 0.090 μm	*M* = 0.773 μm, *SD* = 0.138 μm	83%	Welch’s *F* (1,36.13) = 26.95	<0.0001
Volume	*M* = 0.481 μm^3^, *SD* = 0.170 μm^3^	*M* = 1.295 μm^3^, *SD* = 0.632 μm^3^	37%	Welch’s *F* (1,31.02) = 50.53	<0.0001

### Cell Envelope Thickness

**FIGURE 5:**
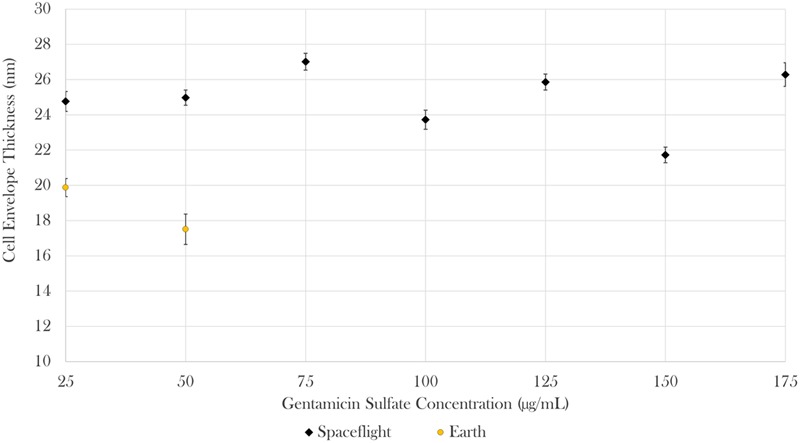
In space, cell envelope thickness increased by 25 and 43% in the 25 and 50 μg/mL samples, respectively. Bars indicate standard error; *n* = 585 and 67 for spaceflight and Earth, respectively.

Cell envelope thickness data was acquired via transmission electron microscopy (TEM). Measurements were acquired for all seven gentamicin concentrations in space (*n* = 585) and for the two lowest concentrations on Earth (*n* = 67). The results showed that envelope thickness varied as a function of drug concentration both in space and on Earth [Brown–Forsythe: *F*(6,508.19) = 22.08, *p* < 0.0001 and *F*(1,26.46) = 5.52, *p* < 0.05, respectively). On average, the cells challenged with 25 μg/mL of antibiotics in space had cell envelopes that were 25% greater than the thickness of their matched Earth controls [Welch’s *F*(1,112.98) = 40.75, *p* < 0.0001; *M*_space_ = 24.76 nm, *SD*_space_ = 4.55 nm, *n*_space_ = 64; *M*_Earth_ = 19.87 nm, *SD*_space_ = 3.67 nm, *n*_Earth_ = 51]. Similarly, the cells challenged with 50 μg/mL of antibiotics in space had cell envelopes 43% greater than the thickness of their matched Earth controls [Welch’s *F*(1,23.08) = 59.75, *p* < 0.0001; *M*_space_ = 24.97 nm, *SD*_space_ = 3.80 nm, *n*_space_ = 78; *M*_Earth_ = 17.51 nm, *SD*_space_ = 3.46 nm, *n*_Earth_ = 16] as described in **Figure 5**. It was not possible to acquire cell envelope data from the 75 μg/mL Earth controls due to the low cell concentrations available in the aliquots from those samples used for this analysis.

### Cell Ultrastructure—Vesicle Formation

**FIGURE 6:**
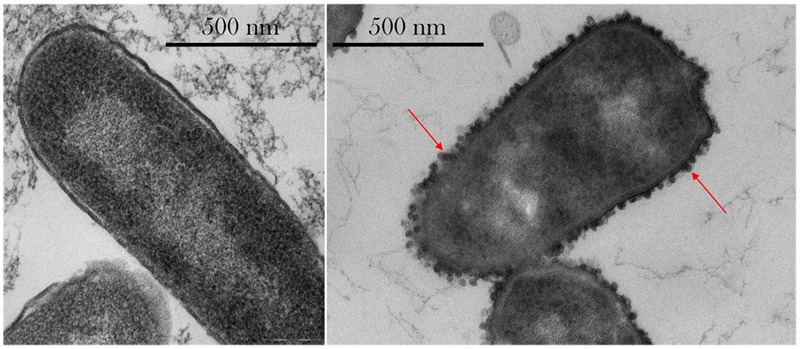
Thin-section transmission electron microscopy (TEM) images of *E. coli*. *Left*: sample cultured on Earth and challenged with 50 μg/mL gentamicin. *Right*: sample cultured in space and challenged with 175 μg/mL shows the presence of extracellular vesicles (red arrows) and irregular cellular shapes. Images taken with a Philips CM 100 TEM at an accelerating voltage of 80 kV.

As seen in **Figure 6**, outer membrane vesicle (OMV) formation was observed in space, especially at the highest concentrations of gentamicin (175 μg/mL). No OMV formation was observed on the Earth samples, as shown in **Supplementary Figure S3**.

### Bacterial Culture Morphology—Cell Aggregation

**FIGURE 7:**
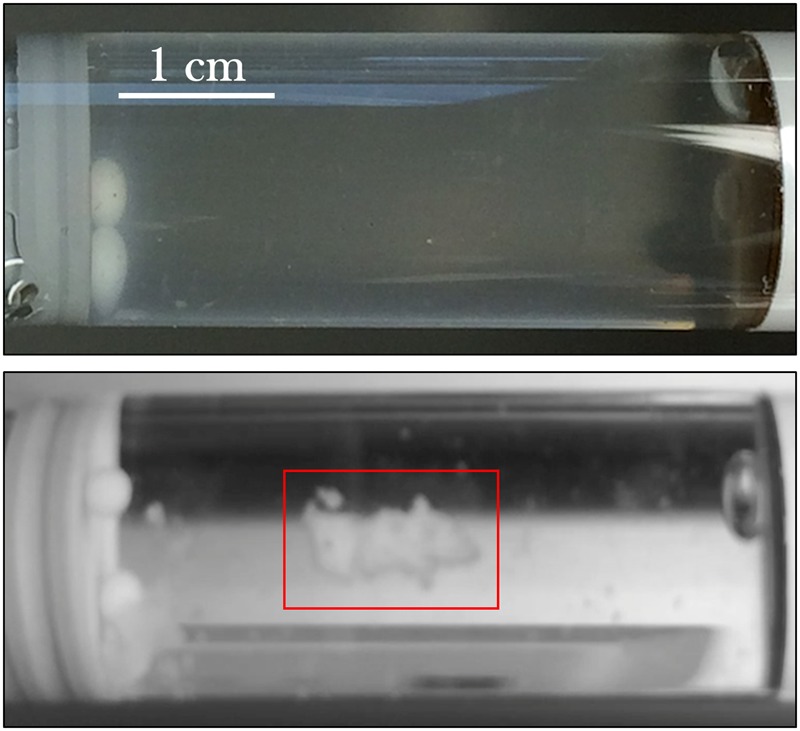
*E. coli* commonly grows with uniform fine turbidity as seen in the 25 μg/mL Earth control (upper image). Spaceflight samples challenged with the concentrations of gentamicin at 125 μg/mL or higher tended to aggregate. The lower image shows the spaceflight sample treated with 175 μg/mL—after its return to Earth—and the clustered cells (red box) in an otherwise visibly clear growth medium.

**FIGURE 8:**
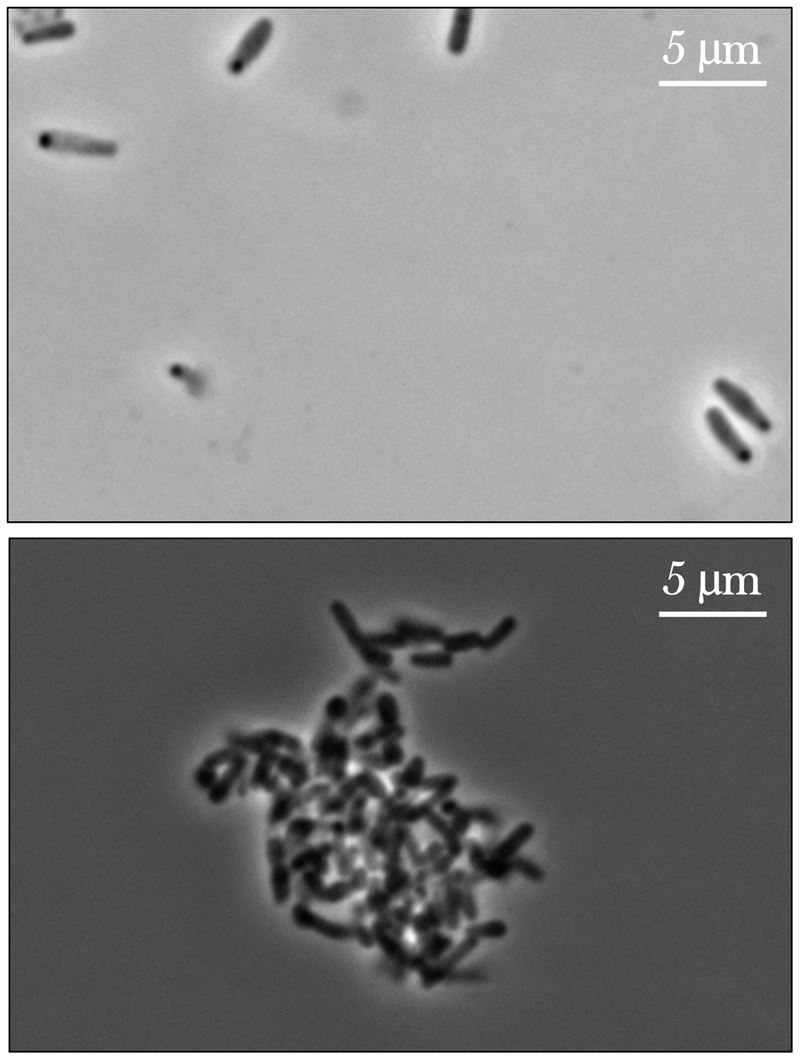
Phase contrast images of *E. coli* cultured on Earth (upper image) and in space (lower image), the latter exhibiting cell aggregation. The upper image shows an Earth control sample challenged with the lowest concentration of gentamicin sulfate (25 μg/mL) and the lower a spaceflight sample treated with the highest concentration of the same drug at 175 μg/mL. The differences in cell size are also noticeable here. Images taken with a Nikon E600 Widefield Microscope.

From initial qualitative assessment of the samples upon their return to Earth, pronounced cell aggregation was the most prominent phenomenon observed. Spaceflight samples with gentamicin sulfate concentrations of 125 μg/mL or higher exhibited cell aggregation to the point that the culture essentially became a contiguous, single cluster as seen in **Figure 7**. This behavior was not observed in the Earth controls and is in stark contrast to the usually uniform fine turbidity cultures observed in 1 *g*. Phase contrast microscopy shows these results more clearly (**Figure 8**).

## Discussion

### Bacterial Growth

No statistically significant difference was observed in the cell population counts that were fixed immediately following inoculation or during the acceleration phase between the spaceflight and Earth cultures. This indicates that the results observed in this experiment are not a result of different starting cell concentrations, and that the initial ratio of antibiotic molecules per bacterium was consistent between spaceflight and Earth controls, respectively. Although the lack of significant difference in cell count during the acceleration phase could also suggest that no changes occurred in lag phase duration, data from only one point in time is insufficient to make such claim, as this does not determine when the acceleration phase actually started. Zea et al. (2016) provided molecular genetic evidence of the “altered extracellular environment” model explaining altered bacterial behavior in space, which would also suggest that quorum sensing molecules may accumulate around the cells and only dissipate to the bulk fluid via diffusion. This may explain the decrease in lag phase duration observed in some spaceflight experiments described by Horneck et al. (2010), which will be discussed in more detail in a separate manuscript. Spaceflight cell concentrations at the end of the growth period were always higher than their respective Earth controls (13-fold increase on average), which was expected based on previous spaceflight observations (as reviewed in Horneck et al., 2010). Furthermore, as statistically indicated in the results, the flight cells were able to grow in the presence of normally inhibitory levels of gentamicin sulfate.

### Cell Size

The average volume of the spaceflight cells was 37% of the Earth controls. The reduction in volume, and therefore surface area and cross-section, translates into a lower probabilistic rate of diffusion-driven molecule–cell interactions. This reduction in contact area is in addition to the presumed altered extracellular environment, which further suggests that molecule–cell interactions will differ in space compared to Earth (Zea, 2015; Zea et al., 2016). The decreased cell size observed in this experiment is in contrast, albeit for different species, with what was reported by Pyle et al. (1999), who noted elongated *Burkholderia cepacia* cells (and chains of cells) in the spaceflight cultures, and with Wilson et al. (2007), who did not see any differences between spaceflight and Earth control *Salmonella typhimurium* cell size or shape. However, both of these strains were motile, and Benoit and Klaus (2007) have already shown a correlation between motility and a potential disruption of the quiescent extracellular environment, thereby eradicating the ensuing proposed effects. Since the *E. coli* strain used in this experiment was non-motile, this suggests that the decrease in cell size may have been a result of extracellular environment factors reducing glucose exposure and increasing acidic level. This is similar to results reported by Jubair et al. (2012), where persister cells from *Vibrio cholerae* exposed to a nutrient-poor condition presented morphologies that were very small in size and with a high degree of aggregation. A decrease in cell size in microgravity has also been reported for a fungal organism: *Candida albicans*. Crabbé et al. (2013) determined that *C. albicans* cultured in space had 70% of the surface area of their matched Earth controls, that they showed a 3D organization (opposite to the flat appearance of Earth samples), and that aberrant forms were observed in space while in 1 *g*, they presented a rounder morphology. This observed decrease in cell size also warrants revisiting mathematical and computational models used to simulate the altered extracellular environment in space, as these earlier efforts assumed that cells were the same size as on Earth (Klaus et al., 1997, 2004; Benoit, 2005; Benoit et al., 2008). Interestingly, as noted by Nanninga (1985), faster growing *E. coli* cells, under certain conditions, were shown to increase in mass. Klaus (1994) suggested—based on optical density data—that *E. coli* in space experienced a slower rate of growth (albeit for a longer duration of time), which was further discussed by Brown (1999). Therefore, the observed decrease in cell size may also be related to a change on growth rate.

### Cell Envelope Thickness

The two experimental sets that could be directly compared indicate that *E. coli* samples cultured in space exhibited an increased cell envelope thickness with respect to their matched Earth controls. This investigation follows at least four spaceflight experiments in which changes to cell envelope thickness have been assessed. The first investigation concluded (although no statistical analyses were presented) that there were no differences in envelope thickness of *E. coli*, but there were on *Staphylococcus aureus* (Zaloguyev et al., 1984; Tixador et al., 1985). Three other studies also reported no changes occurred in cell envelope for *E. coli* (Gasset et al., 1994; Tixador et al., 1994) or *Bacillus subtilis* (Menningmann and Heise, 1994), nor were any differences noted in cell structure in general for *E. coli, B. subtilis*, or *S. aureus* (Juergensmeyer et al., 1999). However, none of these investigations report statistical significance in their analyses, and from all four only one (Gasset et al., 1994; Tixador et al., 1994) reported having fixed the samples in space. In other words, the first three studies measured the cell envelope of cultures that had potentially already re-adapted post-flight to 1 *g* before the samples were processed (Zea, 2015); thus, no appropriate prior spaceflight phenotype is available against which to directly compare our current findings. Interestingly, a thickened cell envelope has been associated with drug-resistant bacterial strains. For example, Raju et al. (2007) and Fukutsuji et al. (2013) separately concluded that gentamicin-resistant *S. aureus* strains showed a thickened cell wall. Similarly, Yuan et al. (2013) showed that amikacin (another aminoglycoside-class antibiotic) resistant methicillin-resistant *S. aureus* presented a thickened cell wall, concluding that this phenotypic phenomenon is associated with adaptive resistance. Nevertheless, no systematic studies on *E. coli*’s cell envelope increase due to aminoglycosides were found in the literature.

### Cell Ultrastructure—Vesicle Formation

Qualitative observations made on the TEM imagery showed the presence of OMVs on the spaceflight samples treated with 75 μg/mL or higher concentrations of gentamicin—with more OMVs observed at the highest concentration tested, 175 μg/mL, but not on the Earth samples. While **Figure 6** shows the difference in OMVs between spaceflight and Earth samples, it is acknowledged that these cannot be directly compared as they represent two different drug concentrations. However, it conveys the observations of OMV formation in space—and not on Earth—and especially at higher drug concentrations. OMVs contain proteins that play a role in the delivery of toxins during infection, biofilm nucleation, defense against antimicrobials, nutrient acquisition, and DNA transfer (Kulp et al., 2015), and are especially helpful in the transport of hydrophobic signaling (quorum sensing) molecules (Mashburn and Whiteley, 2005). Collins (2011) also showed that OMVs can be advantageous coaggregation in biofilm formation. Manning and Kuehn (2011) showed that OMVs can adsorb compounds that act on the cellular outer membrane, including antimicrobial peptides, and hypothesized that vesiculation is a quick response to low doses of stressors before resistance mechanisms can be activated. Kulp et al. (2015) suggested that in wildtype *E. coli*, incomplete lipopolysaccharides structures yielded more OMVs, and that vesiculation levels were inversely proportional to enterobacterial common antigen chain length. McBroom and Kuehn (2007) showed that vesiculation is an independent stress response that enhances bacterial survival rates by allowing them to export stress products—such as damaged or misfolded proteins—and that it is regulated by the level of protein accumulation in the envelope. The observed formation of OMVs at highest concentrations of drug in space, where transport is limited, may indicate that cells were exposed to what on Earth would be considered a “low dose” of drug, which elicited the activation of resistance mechanisms. The low-dose activation under a high-dose environment in space may be an artifact of the altered extracellular environment described by Zea et al. (2016), where a lower rate of drug molecule–cell interaction—exacerbated by a decrease in cell size—was experienced in the microgravity environment.

### Bacterial Culture Morphology—Cell Aggregation

While the Earth and space images on **Figures 7, 8** are not meant to be directly compared—as they represent two different drug concentrations—they convey the qualitative observation of cell aggregation on the spaceflight samples with respect to Earth cultures, especially at higher drug concentrations in space. This qualitative data may also serve as a basis for future quantitative studies. This cell clustering observed on the spaceflight samples is in agreement with previous microgravity studies. Wilson et al. (2007) reported that *S. typhimurium* cultured in space exhibited clear differences in cell aggregation and clumping, which they saw was associated with an extracellular matrix accumulation consistent with biofilm formation. Crabbé et al. (2013) reported *C. albicans* aggregation, enhanced random budding (as opposed to the bipolar budding observed on the Earth samples) and a concomitant differential expression of the genes in this organism’s main flocculation regulatory pathways. On the other hand, Kim et al. (2013) did not observe cell clustering on *Pseudomonas aeruginosa* cultured in space, which they suggested was likely due to differences in the extracellular polymeric substances of the strain they used for their test. Similar results of cell aggregation have been documented from simulated microgravity tests with different model organisms. Self-aggregative biofilm phenotypes of bacterial cultures (*S. aureus* and *P. aeruginosa*) were also reported after growth in rotating wall vessels (Crabbé et al., 2008; Castro et al., 2011). Fungal cultures (*Saccharomyces cerevisiae*) have displayed increased cell clumping under simulated microgravity (Purevdorj-Gage et al., 2006). Similarly, algal (*Chlorella pyrenoidosa*) cell aggregation was observed to increase as a function of time during simulated microgravity [the clumping reported by Mills and Pierson (2003) was strikingly similar to **Figure 8**]; in this case, the authors concluded that the basis for, or significance of this clumping was unknown (Mills and Pierson, 2003). The observed increase in flocculation or cell clustering in space may facilitate conjugation (genetic recombination), which was reported in an early spaceflight bacterial study by Ciferri et al. (1986). The increased cell aggregation of bacteria and fungi observed in space, and its association with biofilm formation and potentially with enhanced conjugation, may therefore present deleterious effects for long-term human spaceflight and warrant further studying of these phenomena.

## Conclusion

A 13-fold increase in final cell count in space was observed with respect to Earth without any statistically significant differences in cell population count at inoculation or when the drug was introduced. This indicates that the higher final cell count achieved in space under normally inhibitory levels of gentamicin sulfate was not an artifact of different starting cell concentrations or cell/antibiotic molecule ratio between Earth and space. Contrast light microscopy and FIB/SEM showed that, on average, cells in space were 37% of the volume of their matched Earth controls. The resultant decrease in surface area and cross section may reduce the rate of molecule–cell interactions in a diffusion-dominated mass transport regime, as is expected to occur in microgravity. This reduction in cellular volume is consistent with other studies where bacteria have been cultured in substrate-poor environments providing further support to the altered-extracellular environment model believed to occur in space (Zea et al., 2016). TEM imagery showed an increase in cell envelope thickness between 25 and 43% in space with respect to Earth. OMVs were observed on the spaceflight cells, especially in the higher drug concentrations cases, but were not seen on the Earth cultures. Both the increase in cell envelope thickness and in OMV formation may be indicative of drug resistance mechanisms being activated in the spaceflight samples. While *E. coli* cultures on Earth were homogenously distributed in the liquid medium through the growth phases, in space they tended to form a single cluster leaving the rest of the medium visibly clear of cells. This cell aggregation may be associated with enhanced biofilm formation reported on other spaceflight experiments and needs to be studied further to determine the cause and significance.

## Author Contributions

LZ, LS, and DK designed the experiment. LZ and LS integrated the experiment for spaceflight and Earth controls. LZ performed the pilot tests and the Earth-component of the experiment and acquired the phenotypic data from light microscopy. LZ and FE performed the statistical analyses. FE and LZ produced the non-microscopy figures and charts. ML and KQ produced the TEM and FIB/SEM imagery and acquired the cell envelope thickness data. LZ, SD, ML, and KQ analyzed the phenotypic data. RM and LS helped with funding acquisition for the Germany-based work and the US-based work, respectively. LZ wrote the manuscript. All authors discussed the results and implications and commented on the manuscript at all stages.

## Conflict of Interest Statement

The authors declare that the research was conducted in the absence of any commercial or financial relationships that could be construed as a potential conflict of interest.

## Supplementary Material

FIGURE S1 A 2 × 10^7^ cell/mL inoculum of *E. coli* was challenged in a gentamicin sulfate dilution series ranging from 80 to 200 μg/mL, and incubated anaerobically for 32 h at 30°C in glass test tubes to replicate the FPAs. The blue boxes indicate the cell concentration at the time of experiment start (2–3 × 10^7^ cell/mL), centered at the average and covering two standard errors in height. While bacterial growth was observed in the no-drug control, no growth was observed as of 80 μg/mL, suggesting the as-tested MIC was between 0 and 80 μg/mL of gentamicin sulfate.

FIGURE S2 A 3 × 10^7^ cell/mL inoculum of *E. coli* was challenged in a gentamicin sulfate dilution series ranging from 10 to 40 μg/mL, and incubated anaerobically for 32 h at 30°C in glass test tubes to replicate the FPAs. The blue boxes indicate the cell concentration at the time of experiment start (3 × 10^7^ cell/mL), centered at the average and covering two standard errors in height. While bacterial growth was observed when challenged with 10 μg/mL and some was debatable at 20 μg/mL, no growth was measured as of 30 μg/mL, suggesting the as-tested MIC was close to 20 μg/mL of gentamicin sulfate.

FIGURE S3 Thin-section transmission electron microscopy (TEM) images of *E. coli* cultured in space, showing no outer membrane vesicle formation at lower drug concentrations (25 μg/mL gentamicin on left, 50 μg/mL gentamicin on center), and multiple (OMVs) at 175 μg/m (right). Images taken with a Philips CM 100 TEM at an accelerating voltage of 80 kV.
