# Supplementary figures and images for: Phenotypic Changes Exhibited by E. coli Cultured in Space
Source: Front Microbiol. 2017 Aug 28;8:1598. doi: 10.3389/fmicb.2017.01598 (PMC5581483; doi:10.3389/fmicb.2017.01598)

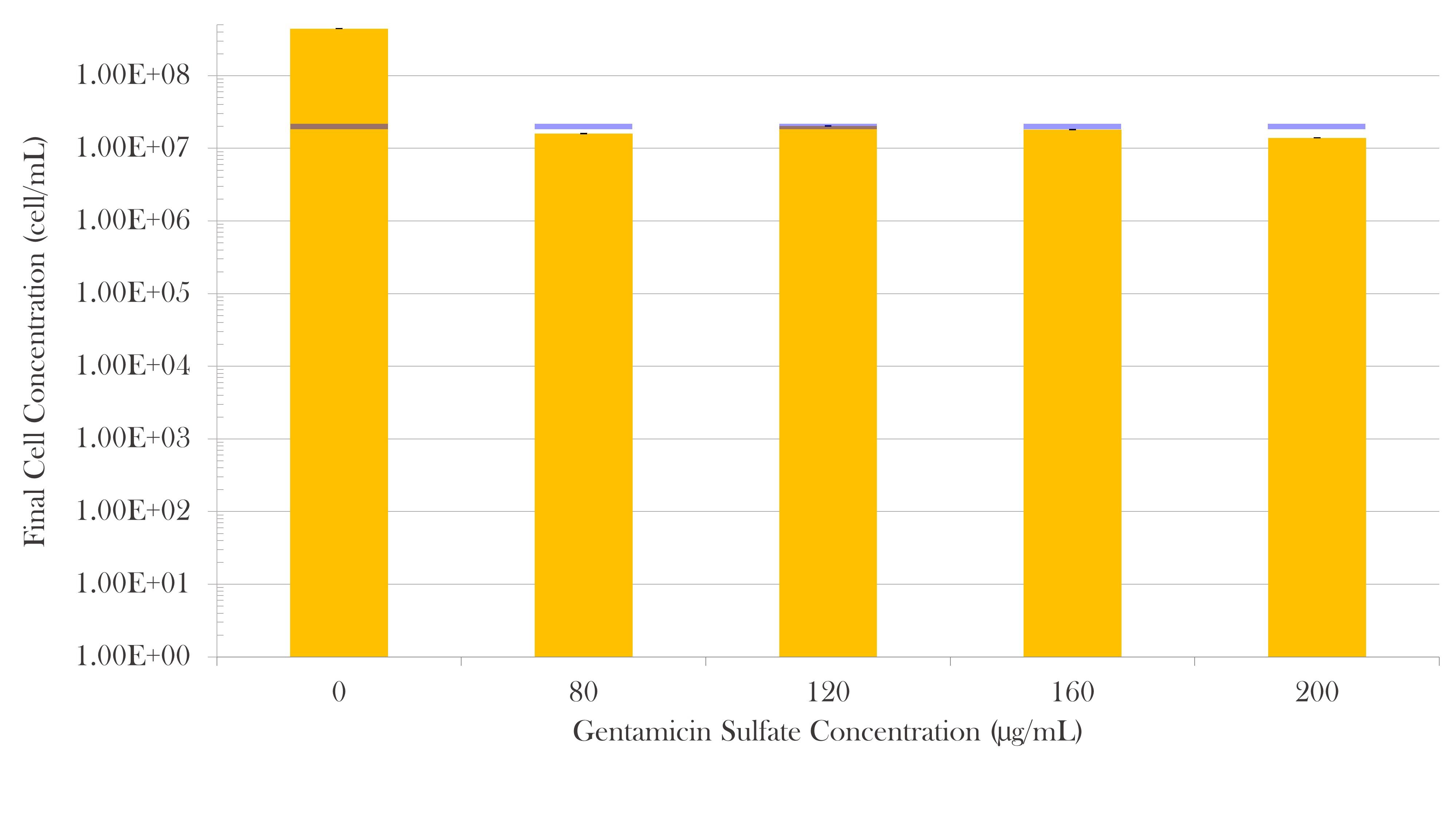

Supplement: FIGURE S1 — A 2 × 10^7^ cell/mL inoculum of E. coli was challenged in a gentamicin sulfate dilution series ranging from 80 to 200 μg/mL, and incubated anaerobically for 32 h at 30°C in glass test tubes to replicate the FPAs. The blue boxes indicate the cell concentration at the time of experiment start (2–3 × 10^7^ cell/mL), centered at the average and covering two standard errors in height. While bacterial growth was observed in the no-drug control, no growth was observed as of 80 μg/mL, suggesting the as-tested MIC was between 0 and 80 μg/mL of gentamicin sulfate. [file Image_1.jpg]

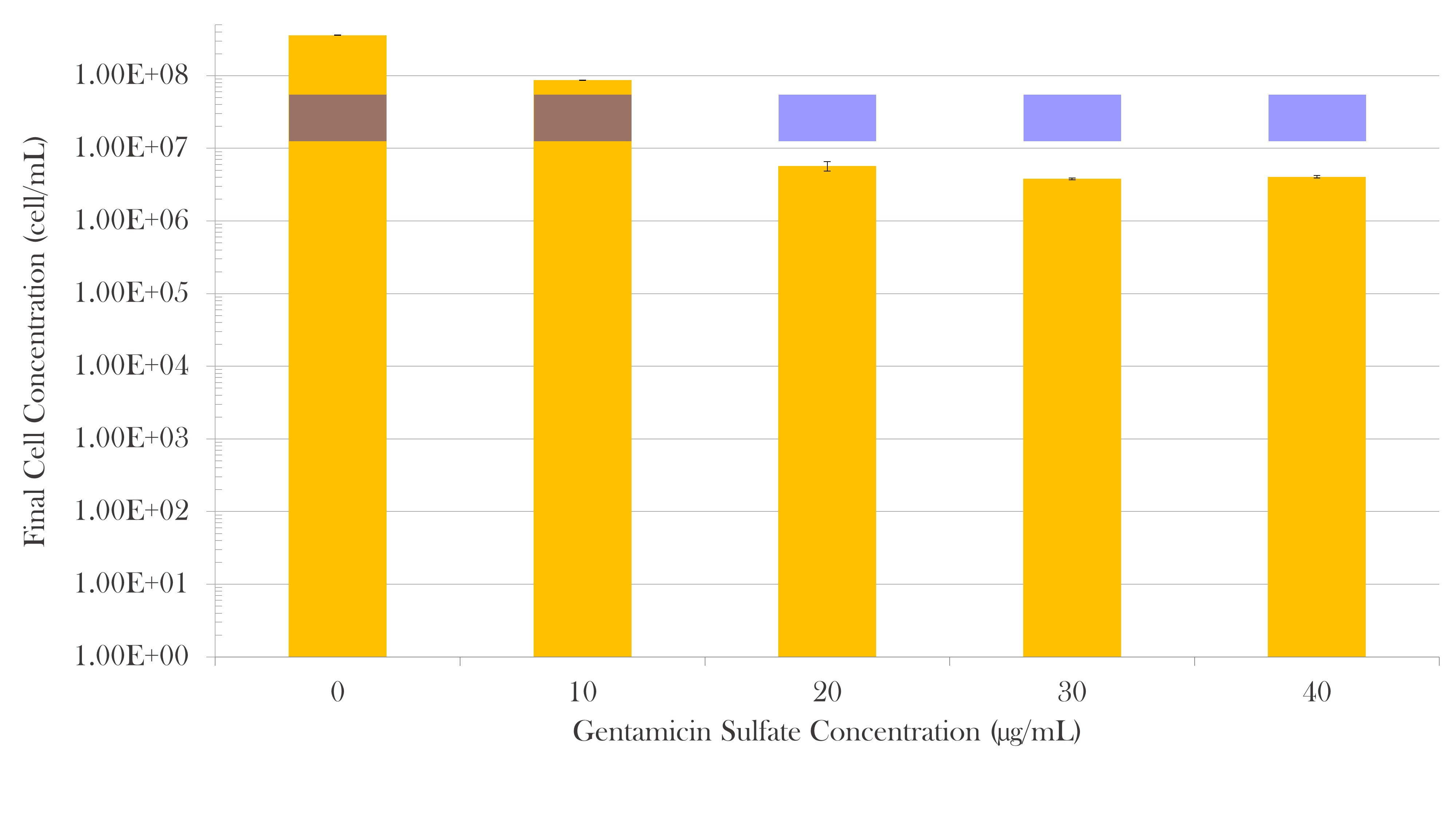

Supplement: FIGURE S2 — A 3 × 10^7^ cell/mL inoculum of E. coli was challenged in a gentamicin sulfate dilution series ranging from 10 to 40 μg/mL, and incubated anaerobically for 32 h at 30°C in glass test tubes to replicate the FPAs. The blue boxes indicate the cell concentration at the time of experiment start (3 × 10^7^ cell/mL), centered at the average and covering two standard errors in height. While bacterial growth was observed when challenged with 10 μg/mL and some was debatable at 20 μg/mL, no growth was measured as of 30 μg/mL, suggesting the as-tested MIC was close to 20 μg/mL of gentamicin sulfate. [file Image_2.jpg]

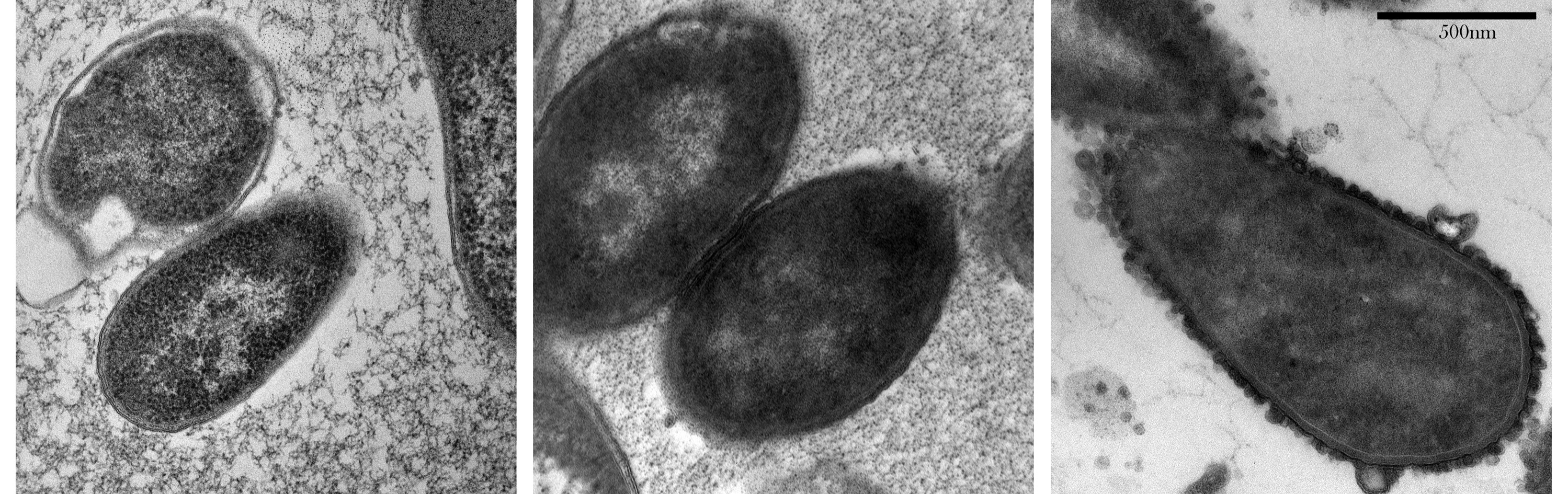

Supplement: FIGURE S3 — Thin-section transmission electron microscopy (TEM) images of E. coli cultured in space, showing no outer membrane vesicle formation at lower drug concentrations (25 μg/mL gentamicin on left, 50 μg/mL gentamicin on center), and multiple (OMVs) at 175 μg/m (right). Images taken with a Philips CM 100 TEM at an accelerating voltage of 80 kV. [file Image_3.jpg]
